# Supplementary material for: Short-Term Thermal Stress Affects Immune Cell Features in the Sea Urchin Paracentrotus lividus
Source: Animals (Basel). 2023 Jun 11;13(12):1954. doi: 10.3390/ani13121954 (PMC10295189; doi:10.3390/ani13121954)
Supplement: Supplementary file 1 [file animals-13-01954-s001.zip › animals-2405894-supplementary.pdf]

# Supplemental materials

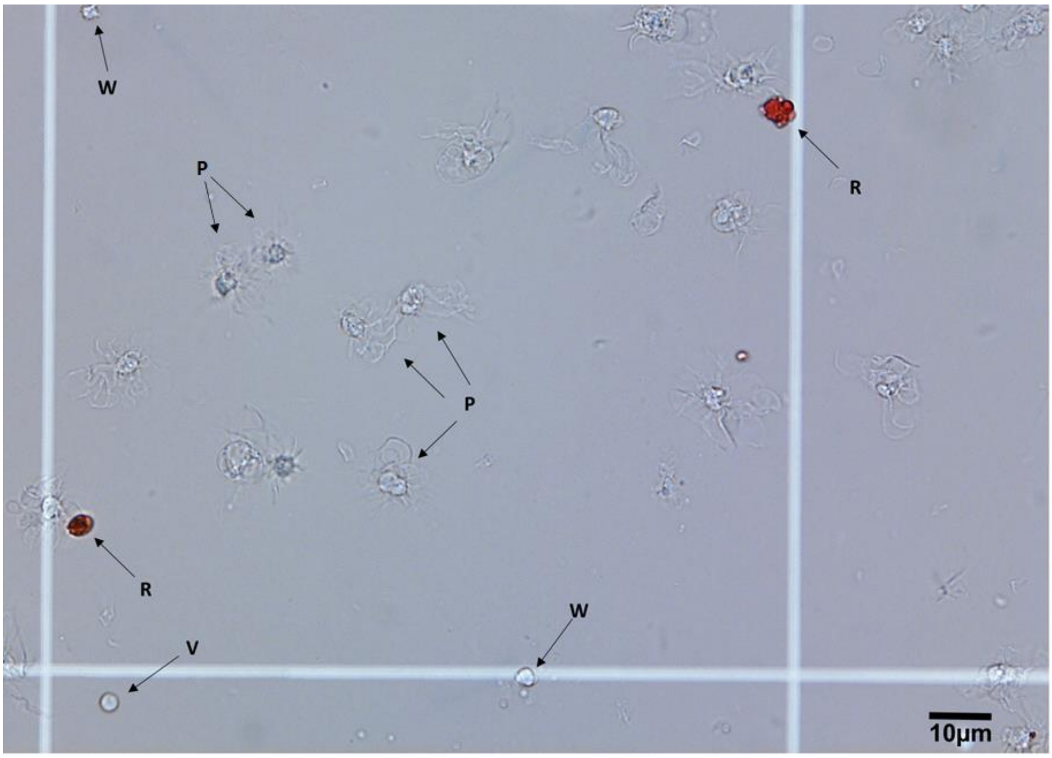


**Figure S1.** *Paracentrotus lividus* coelomocytes in the count chamber. Petaloid and philopodial phagocytes (P), red amoebocytes (R), white amoebocytes (W) and vibratile cells (V) are indicated with arrows.
